# Atonal Music as a Model for Investigating Exploratory Behavior

**DOI:** 10.3389/fnins.2022.793163

**Published:** 2022-06-22

**Authors:** Iris Mencke, Diana Omigie, David Ricardo Quiroga-Martinez, Elvira Brattico

**Affiliations:** ^1^Department of Music, Max Planck Institute for Empirical Aesthetics, Frankfurt, Germany; ^2^Department of Psychology, Goldsmiths, University of London, London, United Kingdom; ^3^Department of Clinical Medicine, Center for Music in the Brain, Aarhus University and Royal Academy of Music, Aarhus, Denmark; ^4^Department of Education, Psychology and Communication, University of Bari Aldo Moro, Bari, Italy

**Keywords:** new music, atonal music, exploratory behavior, predictive processing, aesthetic experience, neuroaesthetics, musical pleasure

## Abstract

Atonal music is often characterized by low predictability stemming from the absence of tonal or metrical hierarchies. In contrast, Western tonal music exhibits intrinsic predictability due to its hierarchical structure and therefore, offers a directly accessible predictive model to the listener. In consequence, a specific challenge of atonal music is that listeners must generate a variety of new predictive models. Listeners must not only refrain from applying available tonal models to the heard music, but they must also search for statistical regularities and build new rules that may be related to musical properties other than pitch, such as timbre or dynamics. In this article, we propose that the generation of such new predictive models and the aesthetic experience of atonal music are characterized by internal states related to exploration. This is a behavior well characterized in behavioral neuroscience as fulfilling an innate drive to reduce uncertainty but which has received little attention in empirical music research. We support our proposal with emerging evidence that the hedonic value is associated with the recognition of patterns in low-predictability sound sequences and that atonal music elicits distinct behavioral responses in listeners. We end by outlining new research avenues that might both deepen our understanding of the aesthetic experience of atonal music in particular, and reveal core qualities of the aesthetic experience in general.

## Introduction

Western art music from the twentieth and the twenty-first centuries is regarded as the continuation of the earlier classic-romantic Western art music tradition and has been characterized by transformation and innovation since its origins in Europe around 1910. The continuous invention of novel compositional techniques and practices has ultimately led to the pluralism of music styles and idioms seen today (Taruskin, 2010). One common element of twentieth/twenty-first century Western art music is its aesthetic premise of creating and presenting something completely novel if not merely experimental (Maletz, 2011; Hiekel, 2016; Mencke et al., 2019). In fact, these aesthetic premises resemble the motivations for engaging with Western art music: one of our studies showed that expert listener’s expectations revolve around the desire to experience both novelty and surprise (Mencke et al., 2022) and similarly, the audience of contemporary art has been found to seek for experiences in which they are presented with novelty and are able to engage with challenging and difficult to understand materials (Gross and Pitts, 2016).

### Fundamental Characteristics of Atonal Music

Despite the tremendous variety of compositional creation in the twentieth and twenty-first centuries, the focus of this article relates to two specific characteristics of this period of music. First, we focus on atonal music (Griffiths, 2001) which we define as music that clearly lacks a tonal center and that suspends tonal hierarchical relations between scale degrees (Bigand and Poulin-Charronnat, 2016). Typically, all 12 tones of the chromatic scale are treated as equal, leading to an abstract and non-hierarchical tonal structure. Atonal music is used here as a style-independent term as it is a description of a certain feature of the music that appeared (and still appears) in various epochs of the twentieth and twenty-first centuries. However, an exemplary compositional style is serialism. It originated in the 12-tone technique developed by Arnold Schoenberg in the early 1920s, and was further developed in the 1950s by composers such as Karel Goeyvaerts, Karlheinz Stockhausen, Pierre Boulez, and Luigi Nono (Dibelius, 1998; Taruskin, 2010). While a large number of pieces composed in a serial manner are strictly atonal, non-serial music that is atonal has been composed throughout the twentieth and twenty-first centuries until today making Atonality a critical feature of many compositions (Kostka and Santa, 2018).

Second, we focus on music that lacks a clear metrical structure. In post-tonal music, perceived rhythms are often highly complex and varied leading to an a-metric structure (Kostka and Santa, 2018). A number of pieces composed during serialism can serve as examples, since the deterministic principles guiding pitch in 12-tone compositions was—among other features—transferred to note duration. Critically, the irregular rhythmic structure and free treatment of meter that results from this compositional technique (Grant, 2001; Kostka and Santa, 2018) ultimately prevents listeners from easily following and entraining with the heard music (London, 2004).

Indeed, both atonal and ametrical musical structures strongly lower the predictability of the music (Kramer, 1989). In particular, serial pieces are, according to Lerdahl, “cognitively opaque,” by which he is referring to the distance between the composed and heard structure (Lerdahl, 1992, p. 115; Lerdahl, 2019). Thus, even though compositions were based on a certain set of principles (e.g., re-presenting a given row of tones reversed and/or turned upside down) for a listener this underlying non-hierarchical organization is barely perceivable. An exemplary series of pieces that resemble or represent both the atonal and the ametric aspects that the current article is targeting are the piano pieces I-IV by Karlheinz Stockhausen, composed in the early 1950s (Stockhausen, 1954; Wörner, 1973). These fall under the category of pointillistic compositions, a technique that has the tendency to “isolate the sounds into ‘points’” (Kostka and Santa, 2018, p. 233) and that generate a “verticalize(d) […] sense of time” (Kramer, 1989, p. 202: for more exemplary pieces see Mencke et al., 2019).

### The Challenge of Atonal Music

With the brain’s prioritization of auditory input containing cognitive reference points (Rosch, 1975) such as the tonic or the cadence in tonal music (Krumhansl and Cuddy, 2010), it is no wonder that atonal music is widely recognized as challenging (Utz, 2016). As early as in the dawn of experimental psychology research, Wilhelm Wundt proposed an inverted U-shaped relationship between stimulus intensity and pleasure, known as the Wundt-curve (Wundt, 1896): a stimulus is liked until a certain level of intensity, but if intensity further increases, pleasure will decay. This theory was extended by Daniel Berlyne, who proposed that each stimulus has a certain potential for physiological arousal: defined as “arousal potential” and referring to the excitement of the nervous system in response to the stimulus (Berlyne, 1971). As a stimulus’ arousal potential is mostly modulated by variables such as complexity, surprise, ambiguity and novelty, atonal music has a high arousal potential that is likely going beyond the pleasure peak for most listeners (Berlyne, 1954, 1970; Marin et al., 2016; Marin, 2020). In turn, listeners with a preference for atonal music might particularly seek for this complexity and therefore potentially have an increased need to engage in cognitive tasks (as can be measured by the *Need for Cognition*—Scale; Cacioppo and Petty, 1982; Cacioppo et al., 1996) in conjunction with a particularly high degree of tolerance to ambiguity (McLain, 2009; for more hypotheses regarding the correlation between preferences for atonal music and interindividual differences see Mencke et al., 2019).

Atonal music typically also has a high degree of dissonance, a key factor underlying the challenge it presents listeners. Dissonant intervals lead to beating and roughness (Helmholtz, 1954) and are often experienced as sensorily unpleasant (Brattico, 2015). It is important to note that dissonance is a central component in perhaps all styles of music and in the context of tonal music, is an important means to generate moments of tension and release (Juslin, 2013; Brincker, 2015; Lehne and Koelsch, 2015; Brattico, 2021), both of which are experienced as pleasurable by many listeners. However, works of atonal music often are predominantly dissonant, which may be why listeners might find this music less appealing. Here, the idea that tolerance toward negative feelings or emotions increases in the context of art perception and that such feelings or emotions are used by artists to reinforce the intensity of an aesthetic experience (Menninghaus et al., 2017) may explain why listeners nevertheless choose to engage with this music. Acceptance of high levels of dissonance in atonal music parallels the fact that feelings of sadness are particularly appreciated in music (Taruffi and Koelsch, 2014; Sachs et al., 2015; Brattico et al., 2016; Eerola et al., 2018).

### Roadmap

In the following, we are concerned with the internal states and cognitive processes that occur while listening to atonal music and argue that in the uncertain environment it provides, neural and cognitive mechanisms associated with exploratory behavior may become engaged. Further we suggest that a focus on this exploratory behavior, may be helpful when considering the predictive processes and hedonic values underlying reception of atonal music. We first give a brief summary of the perceptual and predictive mechanisms that have been suggested to play a key role in music processing and in music appreciation (section “Predictive Dynamics in Music”), before summarizing empirical findings consistent with our proposal that atonal music affords exploratory behavior (section “Neural and Behavioral Levels of Predictive Processing Under High Uncertainty”). In section “Building Predictions in Atonal Music,” we elaborate on how predictive models might emerge as a listener engages with atonal music and argue that these specific model-building processes may be responsible for a sustained exploratory internal state in a listener. Finally, we present future research avenues addressing the role of exploration as a crucial facet of the engagement with atonal music, as well as with respect to aesthetic experience in general (section “Future Research Avenues”).

## Predictive Dynamics in Music

### Musical Expectancy: Bottom-Up and Top-Down

When listening to music, individuals constantly generate expectations about future events and about how the music will evolve. The interplay between the violation and confirmation of these expectations is widely accepted to be a key underlying mechanism for music-induced pleasure in tonal music (Meyer, 1956; Blood and Zatorre, 2001; Huron, 2006). However, in the context of atonal music, where expectations and predictions are more difficult to establish, it seems relevant to have a closer look at the interplay between bottom-up and top-down expectations and how their weighting may differ in atonal music.

With regard to bottom-up expectations Gestalt principles such as pitch and temporal proximity (Deutsch, 1999), rhythmic grouping (Koelsch, 2012), and sound similarity (Bregman, 1990; Deutsch, 1999) have been proposed to be central. According to Lerdahl, grouping preference rules can additionally be based on changes in intensity and articulation (Lerdahl and Jackendoff, 1983; Clarke, 1999). In other words the grouping of notes or phrases into processable chunks can be carried out with regard to a number of musical properties. Bottom-up grouping is thought to happen in early processing stages, namely in short-term memory comprising a time span of 250 ms–8 s (Snyder, 2008). Furthermore, grouping processes in music are related to chunking which determines the memorability of sequences, whereby the better a musical phrase can be chunked, both the better it can be recalled (Lerdahl and Jackendoff, 1983; Bregman, 1990; Snyder, 2000) and the more efficiently it can be processed (Deutsch, 2013).

While, on the one hand, our expectations are strongly shaped by Gestalt-like principles, thanks to bottom-up grouping, musical expectancy is similarly modulated, on the other hand, by top-down mechanisms that are linked to statistical learning (Loui and Wessel, 2006; Pearce, 2018). Our brain is sensitive to auditory regularities in the environment, internalizing and using them to predict future events (Friston, 2009; Clark, 2013). In the case of music, this means that the predictive model of the musical style(s) that we grew up with is the one that we internalize most (Kliuchko et al., 2019) and for which we have the best predictive model. Processes underlying musical expectancy are not only modulated by cultural background but also by style-specific expertise and by piece-specific knowledge (Huron, 2006).

These bottom-up and top-down expectations stemming from both Gestalt principles and statistical learning have been shown to work in parallel and be critical to the reception of tonal music (Morgan et al., 2019). In atonal music, however, as we will argue in later sections of the article, the difficulty of the listener to apply clear top-down expectations may mean an upweighting of bottom-up grouping effects.

### Predictive Coding of Music

One theory that combines bottom-up and top-down processes is the theory of predictive coding and having shown great success in accounting for how we respond to tonal music, it is relevant to consider it in the context of atonal music.

The predictive coding theory is based on the assumption that the brain, as a prediction machine, continuously tries to predict upcoming sensory input by means of generative cognitive models on higher levels. These top–down predictions encounter bottom-up sensory input and in cases where the model’s prediction is incorrect, a prediction error signal occurs to update the model (Friston and Kiebel, 2009; Clark, 2013). To measure this prediction error signal, often times the mismatch-negativity (MMN) is used, which is a brain response to deviating sounds in a regular sound environment (Näätänen et al., 2007). The MMN is suggested to index violations of predictions that are set up by the statistics of an unfolding melody (Denham and Winkler, 2006) and is therefore regarded as a neural marker of prediction error in musical processing.

Critically, research shows that the brain effectively adapts to the statistics of incoming input and thereby to the level of predictability of the sensory input. In high uncertainty contexts, predictions therefore become attenuated or imprecise and the error minimization process is consequently reduced, if not nullified (Garrido et al., 2013; Sohoglu and Chait, 2016; Heilbron and Chait, 2018). Accordingly, it has been suggested that high predictive uncertainty of a stimulus is reflected in a weak predictive model (Vuust and Frith, 2008; Ross and Hansen, 2016; Koelsch et al., 2019) and that in such high-uncertainty conditions imprecise prediction errors have been suggested to be “effectively ignored” (Koelsch et al., 2019).

Recently, the predictive coding framework has been used to account for the enjoyment that humans derive from listening to music (Vuust and Kringelbach, 2010; Koelsch et al., 2019; Brattico, 2021). In particular, it was put forward that—since music plays with a listeners’ expectations and predictions—music provides the opportunity to constantly resolve uncertainty (Koelsch et al., 2019). It has been suggested that the enjoyment of music stems from the interplay between levels of predictability that are typical for music, and how those unfold over time. In some moments, music allows us to generate strong predictions, and at other moments, our predictions are more uncertain, with such changes in the precision of predictions relating to a range of different musical properties such as to meter, melody, rhythm of harmonic progressions (Pearce and Wiggins, 2012; Koelsch et al., 2019).

In essence, the process of resolving uncertainty is proposed to be an appealing element when listening to any music that has some degree of regularity and crucially, resolving uncertainty is typical for states of exploration (Friston, 2010; Schwartenbeck et al., 2013). However, the extent to which a drive to resolve uncertainty is important in atonal music has yet to be fully explored.

## Neural and Behavioral Levels of Predictive Processing Under High Uncertainty

Generally, atonal music has indeed long been a fringe topic in the literature of music cognition, with Western tonal music being central to most studies. The atonal music studies that do exist make explicit listeners’ everyday intuitions that atonal music is more difficult to recognize and remember than tonal music (see for instance: Cuddy et al., 1981; Dibben, 1994; Dowling et al., 1995; Schulze et al., 2012). An interesting question is what insights a predictive coding framework can offer the study of atonal music. Indeed, as predictive coding frameworks have predominantly dealt with Western tonal music, their application to atonal music would seem to present a particular challenge: this both when considering predictive processes in general and when considering the role predictive dynamics might play in music-induced pleasure.

### Prediction in Atonal Music

Only a few studies have focused on the extent to which listeners make or do not make predictions when listening. In a series of experiments, Krumhansl et al. (1987) provided data suggesting that tonal predictions are being made even when individuals (from Western culture) listen to atonal sequences. After hearing a 12-tone row, trained musicians expected that the following tone would not have been heard in the preceding row and would not suggest any sort of tonal center. This is consistent with the interpretation of another study (Ockelford and Sergeant, 2012) suggesting that listeners’ expectations in response to atonal music may follow an “anti-structure” and the claim that listeners of atonal music adapt to the unpredictability of the music by “expect(ing) the unexpected” (Huron, 2006, p. 331). In any case, atonal contexts have been shown to behaviorally evoke weaker expectancies than a major-minor tonal context with higher false alarm rates for recognizing atonal unexpected target notes (Vuvan et al., 2014). All of the above accord with the idea that atonal contexts provide a more significant challenge to a listener than a major-minor tonal context even though studies could provide evidence that a listener is implicitly internalizing other regularities than pitch as for instance with regard to timbre (Tillmann and McAdams, 2004). In recent work, we asked how high-uncertainty musical contexts affect brain activity, in particular the precision of automatically generated predictions (Quiroga-Martinez et al., 2019; Haumann et al., 2021). Consistent with research showing that early evoked responses are dampened in unpredictable contexts (Garrido et al., 2013; Hsu et al., 2015; Sohoglu and Chait, 2016; Southwell and Chait, 2018), we provided evidence that contextual uncertainty can attenuate a pre-attentive neural response to deviating sounds known as the mismatch-negativity (MMN) (Quiroga-Martinez et al., 2020a,b). However, in a yet more recent study, we demonstrated that contextual uncertainty may not always completely eliminate sensory sensitivity to deviating events (Mencke et al., 2021). In contrast to previous studies that largely used musical stimuli composed according to Western tonal rules, we created atonal melodies based on original 12-tone rows by Arnold Schoenberg and then measured how the brain responded to deviants using the MMN (Näätänen et al., 2007). In 20 non-musicians measured with magnetoencephalography (MEG) and other 39 non-musicians tested behaviorally, we found that, while the MMN response to four types of deviants (pitch, timbre, intensity, location) did not differ between tonal and atonal sequences, the behavioral accuracy and confidence in pitch deviance detection were nevertheless significantly lower in atonal sequences (Mencke et al., 2021).

Taken together, our results show that subjective ratings, which reflect processing stages in which conscious awareness is engaged, are strongly affected by the atonal structure of the stimuli even when earlier sensory processing stages addressed by the MMN may remain relatively unaffected. We regard this as evidence of a dissociation between sensory and cognitive musical expectations, whereby the latter may be most affected in the context of high uncertainty stimuli (see also: Neuloh and Curio, 2004). An interesting possibility is that the lack of accuracy and confidence that accompanies listening to atonal music enhances the adoption of searching or exploratory listening behaviors in pre-disposed listeners.

### The Phenomenal Level: Listening Experts and the Experiential Dimension

Previous work shows that listeners’ predictions are weaker for tonal than atonal music (Vuvan et al., 2014). Our recent data further demonstrate that processes in which conscious awareness is involved may be particularly affected by the lack of a tonal hierarchy (Mencke et al., 2021). However, relatively little is known about the phenomenal experience of atonal music. Specifically, characterizations of the nature of aesthetic experiences of atonal music, the key affective dimensions underlying engagement with this music, and the sources of enjoyment reported when listening to it, all remain largely absent.

To fill this gap, a series of interviews with experts specialized in atonal music and, by way of comparison, listening experts from the field of classic-romantic music, were conducted by the first author (Mencke et al., 2022). The aim of the study was to investigate a variety of experiential dimensions underlying an aesthetic experience with atonal music as well as to explore hedonic values, appreciation and pleasurable experiences with this music. Sixteen interviews were conducted with 8 experts in each group and the interview guide (for both groups) comprised questions about several aspects of a listening experience with the corresponding style of expertise. Physiological, cognitive and affective dimensions were covered. After the transcription of audio-recorded interviews, the textual material was analyzed qualitatively both following a deductive and an inductive step (Mayring, 2014).

The analysis revealed striking differences between the expert groups regarding how they described engagement as well as pleasurable experiences with music from their style of expertise. In the following we elaborate on a few findings of the analysis of the reports of the atonal music group that were most frequently mentioned.

First, the notion of exploration was prominent in many of the resulting themes of the qualitative analysis. Participants from the atonal music group repeatedly reported on their adoption of an exploratory attitude and on the fact that they enjoyed the active exploration of a piece of music. With regard to the latter, they emphasized their appreciation of the opportunity to continuously seek new ways of engaging with a piece of music by “probing it through listening.”

Second, the results showed that pattern recognition, i.e., the perception of a memorable musical motive or phrase, may be a large source of pleasure when listening to atonal music. Specifically, when probed regarding experiences of beauty and pleasure during listening, participants reported the joy experienced when discovering a certain pattern in the music (“joy of discovery”) and the high valuation of such moments of perceptual insight. One participant called this insight a “listening guide,” something that leads one through a piece of atonal music. According to the participant’s descriptions, such patterns were, for instance, related to a certain rhythmic structure that re-occurred from time to time. Thus, pleasurable moments were shown to emerge from recognition of an underlying pattern or structure in the music. Another finding supporting this notion are reports that increasing coherence as well as the confirmation of one’s own expectations was experienced as highly positive.

Third, participants from the atonal group emphasized their adoption of an open stance and of their emergent feelings of curiosity. Here it is important to note that, while it has frequently been suggested that the curiosity and openness at a trait level correlate with a preference for (particularly complex) art (Feist and Brady, 2004; Silvia, 2008; Nusbaum and Silvia, 2011; Omigie, 2015), the relevance of state curiosity and openness during engagement with music has received only very little attention in empirical research. In one study employing a continuous rating methodology, it was shown that the perception of change while listening to music can lead to increases in feelings of curiosity as to how the music will unfold (Omigie and Ricci, 2021). Interestingly, in another recent study (Omigie and Ricci, 2022) a difference in the way state curiosity emerges in high- vs. low-uncertainty contexts was demonstrated, whereby while in low uncertainty contexts, high information content notes tended to induce curiosity, in high uncertainty contexts, both low and high information content events were able to drive curiosity. While that study did not focus on listeners that are experts in atonal music, it is interesting to consider how it aligns with the idea that the feeling of discovering a pattern (encountering a predictable event in a high entropy context) can lead especially engaged (atonal music) listeners to feel greater curiosity and further inclinations to explore the heard music as it unfolds.

Taken together, recent empirical data suggest that the complexity in atonal music may make listeners desire both coherence and a decrease in complexity, a phenomenon that is clearly in line with Berlyne’s arousal theory proposing an inverted U-shaped relationship between hedonic value and the complexity of a stimulus (Wundt, 1896; Berlyne, 1970, 1971) and is further supported by other neuroscientific and behavioral studies in the context of empirical music research (Cheung et al., 2019; Gold et al., 2019). Critically, contrary to what was reported by the atonal music experts in the interview study (Mencke et al., 2022), a key role for pattern discovery was at no point mentioned by the classical music group. Rather, these listeners instead reported enjoying the clear structure of classical music, an interesting harmony or rhythm, and the “thrill of an unfamiliar interpretation” of a familiar piece (reports on the entire data see Mencke et al., 2022).

In sum, we argue that the following interrelated processes may be critical to the aesthetic experience of atonal music: The exploratory stance, by promoting the identification of sensory and perceptual features conveying coherence, may allow state curiosity to emerge. In turn, state curiosity by encouraging further engagement increases the opportunity for moments of structural insights to emerge (Brattico, 2015). This oscillation between exploring and moments of structural insights may characterize the experience of atonal music.

## Building Predictions in Atonal Music

Having considered the evidence for an exploratory stance during atonal music listening, the current section elaborates on the mechanisms underlying the interplay between this stance and momentary phases of structural insight and on how predictive processes may give rise to positive hedonic values during online processing of atonal music.

### Grouping and Gestalt Processes

As described above, memorable patterns can emerge in atonal music, for instance, when a repetitive structure arises in a piece based on pitch, rhythm, timbre, pitch, or loudness similarities. Critically, the recognition of memorable patterns, which constitutes moments of insight or a “listening guide,” may indicate the cognitive process by which a Gestalt or a grouping—and therefore a momentary predictive model—has been developed.

Indeed, it is likely that model-building processes in the context of atonal music are strongly related to grouping principles and Gestalt heuristics (Bregman, 1990; Deutsch, 1999). That low-level predictive models are indeed present in an atonal context (Mencke et al., 2021) supports the assumption that low-level musical features may be used to produce those moments of structural insight, even when they do not reach conscious awareness. A listener of an atonal music work—more than a listener of tonal music—may have to rely on such bottom-up and implicit perceptual principles related to pitch proximity, rhythmic grouping or sound similarity (Bregman, 1990; Deutsch, 1999; Koelsch, 2012), as well as neural processes such as stimulus specific adaptation and forward masking. Sound similarity, as an example, would provide the listener an opportunity to perceive a certain instrumental group in an ensemble piece as a single Gestalt or, in terms of auditory scene analysis, as one sound stream. Another example—for instance, considering one of the early piano pieces by Stockhausen (1954)—would be a few subsequent notes that are played in the high register while being preceded and succeeded by melodies in a very low register: here a chunk or a Gestalt would be perceived based on pitch proximity. When and how Gestalts or chunks emerge and are recognized is modulated by a listener’s musical and cultural background, their style-specific expertise, their piece-specific knowledge, and by cognitive abilities such as working memory capacity (Snyder, 2000; Tillmann and McAdams, 2004; Huron, 2006). By influencing the ways in which Gestalts, and thereby low-level and sensory predictions, are generated, all of these may be expected to modulate the experience of atonal music.

In sum, even though Gestalt elements are typically avoided in atonal music, growing evidence suggests that Gestalt-related processes of pattern identification and recognition should be taken into account when considering how atonal music is received. The lack of clear, familiar Gestalts in atonal music, and the complex environment that it presents to a listener, results in attention being enhanced in those moments in the music when regularity increases (Jones, 2019) or when sound events are particularly salient.

### Structures of Saliency

Gestalt-related processes may be linked to so-called salient events in the context of listening to atonal music (Lerdahl, 1989). Lerdahl argued that listeners of atonal music “grab on to what they can: relative salience becomes structurally important” (Lerdahl, 1989, p. 84) and indeed, the relative salience of musical events (indexing structural importance) has been shown to be more relevant for listeners of atonal music than for those of tonal music (Deliège and Mélen, 1997; Dibben, 1999).

It has been suggested that such salient musical features function as cues, serving as “memory triggers” in situations in which they are repeated (Daynes, 2011). Interestingly, listeners have also been shown to remember and localize certain excerpts in atonal pieces if they can relate them to a specific cue (Deliège, 1989). Thus, salient cues that elicit an increase in attention and awareness could be the basis for building a memory trace of an atonal piece thanks to the opportunity they provide for the listener to form a perceptual chunk (Jones, 2015).

Salience can be generated in different ways (for instance by rhythmic, timbral or dynamic means). These structures of saliency could form so-called “event hierarchies” (Bharucha, 1984; Deutsch, 1984) and may conspicuously vary throughout a single work of music so that only provisional musical hierarchies can be generated (Imberty, 1993; Ordoñana and Laucirica, 2017). Such momentary hierarchies may only be applicable to certain phrases or parts within a piece and are therefore “extremely fluid for the hearer” (Imberty, 1993, p. 331) inasmuch as they do not have a fixed or a definite structure. Only temporarily valid predictive models can be built: one chunk that was built in a certain phrase of a musical work and that provided a momentary stability and a momentary predictive model might not be applicable in the next phrase of a work.

We therefore suspect that the generation of predictive models in the context of listening to atonal music is a highly transitory and fluid process, characterized by only temporary stability and temporary moments of increased predictability. It requires a high degree of adaptability of the listener and thus prompts the continuous exploratory behavior that is typical for an aesthetic experience with atonal music.

### Exploration in Atonal Music

Taking into account the reports from dedicated and professional atonal music listening experts about the adoption of an exploratory state even after many years of exposure (Mencke et al., 2022), and considering the deliberate goal of avoiding predictability, particularly in serialist compositions (Stockhausen, 1963; Boulez, 1972; Kramer, 1989; Lerdahl, 1992; Hiekel, 2016), we suggest that atonal music is an artistic language that, more than other musical styles, affords its listeners an exploratory attitude.

Exploratory behavior is essential in environments that are novel, have surprising elements, and are complex (van Lieshout et al., 2020). Humans intrinsically seek knowledge (Berlyne, 1954; Perlovsky, 2010), have a drive for curiosity (Jepma et al., 2012), and actively engage with novel environments (Kidd and Hayden, 2015). Exploratory behavior can be seen as resulting from a desire to reduce uncertainty, a mechanism that all biological agents share (Friston, 2010; Schwartenbeck et al., 2013). Some studies and theoretical proposals suggest that uncertainty reduction is linked to positive affect and argue that it allows an agent to either confirm or update their existing predictive models (Van de Cruys, 2017; Koelsch et al., 2019; Kraus, 2020). In the case of atonal music, this may be particularly relevant since increased regularity in these uncertain environments are reported to be particularly pleasant (Mencke et al., 2022). A recent study using musical stimuli has shown that this model update is linked to activity in the nucleus accumbens (Gold et al., 2019), a central part of the dopaminergic mesolimbic reward pathway (Koelsch, 2014) and known to index pleasurable emotional peak experiences (Salimpoor et al., 2011). The hedonic value underlying atonal music might be closely linked to the positive affect stemming from a momentary reduction of uncertainty.

While exploration can be defined as “learning about the properties of an uncertain environment” (Gazzaniga et al., 2010, p. 1,065), a complementary behavior termed “exploitation” refers to a state in which an individual benefits from a familiar environment in which they know where rewards can be obtained (Kidd and Hayden, 2015). Arguably, Western tonality, with its inherent tonal and metrical hierarchy, offers a (Western) listener the opportunity for immediate exploitation (Meyer, 1956; Huron, 2006; Vuust and Frith, 2008; Rohrmeier and Koelsch, 2012; Salimpoor et al., 2015; Koelsch et al., 2019). In contrast, for atonal music, which lacks such a fundamental predictability there is no structure to be gleaned —especially when we first listen—, and accordingly no immediate exploitation to be afforded.

Rather, we argue, atonal music, by being minimally predictable, affords a mode of exploration that allows brief pleasurable moments of insight to emerge but these insights may disappear as quickly as they emerge. This exploratory state is likely to be highly relevant for a number of different sorts of engagements with art, from abstract painting and contemporary dance to modernist poetry (Saklofske, 1975; Cupchik and Gebotys, 1990). It might additionally be relevant for many other genres from twentieth to twenty-first century art music that present listeners with unconventional musical structures. Accordingly, the minority of individuals who are willing to engage in atonal music are likely also willing to engage with these kinds of artistic languages (Mencke et al., 2019). Finally, an exploratory state might also be adopted if a listener engages with music from an unfamiliar culture as for instance when Western listeners encounter music based on the pentatonic scale.

## Future Research Avenues

The constant generation of new predictive models facilitated by atonal music may provide unique insights, not only with respect to how a psychological state of listening to atonal music can be characterized, but also with respect to aesthetic experiences more generally.

A first issue pertains to the role of pattern discovery in music and other cross-modal aesthetic domains and its relation to the hedonic value. The cognitive mechanism related to the successful recognition of a musical pattern could be regarded as the auditory analog of the “Aesthetic Aha” in the visual (art) domain, which refers to a pleasurable moment stemming from pattern recognition (Muth and Carbon, 2013; Graf and Landwehr, 2017; Muth et al., 2018). It has been argued that the pleasurable effect originates in the sudden increase of processing fluency (Topolinski and Reber, 2010), a cognitive process that has been associated with liking (Reber et al., 2004). This effect is corroborated by studies showing that moments of insight evoke intense positive feelings (Shen et al., 2016; Webb et al., 2018). With regard to problem solving or verbal comprehension, the cognitive process of insight is regarded as an unexpected solution for a problem (Subramaniam et al., 2009) and a moment of sudden comprehension (Bowden et al., 2005; Kounios and Beeman, 2009). In one study investigating verbal problem-solving, insight moments were shown to be reflected in an increase in synchronous gamma-band oscillatory brain activity in the right anterior superior temporal gyrus, that was preceded by an increase in alpha-band activity at the right occipital cortex (Jung-Beeman et al., 2004). The authors suggested that these electrophysiological signatures point to the transition from a pre-attentive to an attentive state and reflect the “conscious availability of a solution” (Jung-Beeman et al., 2004, p. 506). In a more recent study that used auditory sequences, it was shown that the shift from a random to a regular sequence was accompanied by a sustained increase in the amplitude of neural signals (Barascud et al., 2016; Sohoglu and Chait, 2016; Southwell et al., 2017).

For this reason, it would be interesting to study whether such moments of insight similarly emerge in the context of music listening, particularly in the context of high-uncertainty music such as atonal music. Here, such moments might potentially emerge when an auditory object is perceived (Winkler et al., 2009), which could lead to an increase in conscious auditory perception or auditory awareness (Gutschalk et al., 2008; Dykstra et al., 2017). In order to study this, a series of musical stimuli could be created in which the complexity is gradually modified, either with regard to pitch or meter. While simpler stimuli would offer many ways to form chunks, the more complex stimuli would hamper the formation of chunks or auditory objects. Using magneto- or electroencephalography (MEG/EEG), brain responses could be analyzed with the aim to see whether neural activity evolves in comparable patterns such as those found for verbal moments of insight. Complementary data collection of ratings addressing (a range of) questions related to liking or preferences would shed light on any relationships to hedonic values.

Second, the predominance of exploration afforded by atonal music opens up the opportunity to study effects that stem from an aesthetic attitude—a state of mind that, according to philosophical aesthetics, “is entered into, voluntarily and consciously, by an individual, making that individual receptive to having an aesthetic experience” (Fenner, 1998, p. 1954) and thereby involves a certain intentional stance of the listener (Fenner, 1996, 1998; Kemp, 1999; Levinson, 2009; Brattico and Pearce, 2013; Hodges, 2016). Some scholars conceptualize this stance as an attentional focus on the formal and perceptual properties of an object in the context of an aesthetic experience (Levinson, 2009; Juslin and Isaksson, 2014). This is in line with the conceptualization of an aesthetic experience as a person’s phenomenal state while consciously immersing and interacting with the music and in which the attention is directed to the music’s perceptual and formal properties as well as its cognitive and affective interpretations (Brattico and Pearce, 2013; Wald-Fuhrmann et al., 2021). It has been proposed that an aesthetic attitude entails a focus on “sensory impressions, based on low-level features of the music” (Juslin, 2013, p. 248), i.e., features of the music that potentially serve as important cues in order to build Gestalts. Reybrouck (2015) proposes that art contexts force an individual to explore the content of an artwork, which may, in turn, cause this focus on sensory or perceptual properties. One conception and operationalization of the aesthetic attitude might therefore be that it evokes a particular attentional focus on sensory and perceptual properties of the music.

The empirical findings summarized in chapter three indeed support the idea that a focus on perceptual properties is afforded by atonal music, and thereby corroborates the proposal that “some pieces of music will “invite” an aesthetic attitude to a greater extent than other pieces (because of certain formal features)” (Juslin, 2013, p. 247). Atonal music might therefore be helpful when studying behavioral and neural correlates of an aesthetic attitude. In an experiment, one could utilize different musical stimuli with varying degrees of predictability and complexity (for instance, classical music vs. jazz music vs. atonal music). In order to investigate whether the attentional focus shifts as a function of complexity, participants’ responses to questions relating to a number of different low-, medium-, and high-level features of the music could be collected. Based on what presented above, we would predict a positive correlation between degree of complexity and attentional focus on low-level properties of the music. By contrast, in music that provides a certain structure and clear anchor points, such as a tonal hierarchy, the attentional focus on sensory properties would remain in the background, i.e., such basic properties would remain subconscious.

Here it is worth noting an interesting alternative strategy for dealing with uncertainty that was described by the interviewed participants: namely, the adoption of an attitude in which they tried to avoid an analytic and structured listening mode and instead switched to a more free, open-ended listening mode. One respondent said: “If you go in without any expectation of understanding and just let yourself be affected, then you end up understanding more than if you had gone in already expecting to acquire knowledge.” Thus, the acceptance or awareness of not being able to fully predict and ultimately to exploit the music for an open-ended exploratory listening experience might be another, complementary strategy of how to deal with the perceptual challenge present in this music. This might lead to the generation of subjective meaning that goes beyond the positive experience of successfully generating or refining predictive models. Thus, how this particular aspect of “guidedness” can be conceptualized, what it underlies and which effects it has, and ultimately how this mode of open-ended exploration interacts with a mode of active exploration, represent another novel potential research avenue.

## Conclusion

In this article we aimed to provide a novel perspective on how an aesthetic experience with atonal music could be characterized. With reference to neural and behavioral evidence, we emphasized that an exploratory state is important and often adopted given that it facilitates the discovery of novel reference points. Importantly, this state becomes particularly crucial in relation to the poor prospects of exploitation. Future research that aims to study the exploratory state in the context of an aesthetic experience should therefore include atonal music in their research paradigms.

## Author Contributions

IM generated the idea and hypothesis for this article and wrote the first draft of the manuscript. All authors equally contributed to the further development and refinement of the argumentation and to writing and revising the article.

## Conflict of Interest

The authors declare that the research was conducted in the absence of any commercial or financial relationships that could be construed as a potential conflict of interest.

## Publisher’s Note

All claims expressed in this article are solely those of the authors and do not necessarily represent those of their affiliated organizations, or those of the publisher, the editors and the reviewers. Any product that may be evaluated in this article, or claim that may be made by its manufacturer, is not guaranteed or endorsed by the publisher.

## References

[B1] Barascud N. Pearce M. T. Griffiths T. D. Friston K. J. Chait M. (2016). Brain responses in humans reveal ideal observer-like sensitivity to complex acoustic patterns. *Proc. Natl. Acad. Sci. U.S.A.* 113 E616–E625. 10.1073/pnas.1508523113 26787854 PMC4747708

[B2] Berlyne D. E. (1954). A theory of human curiosity. *Br. J. Psychol.* 45:180. 10.1111/j.2044-8295.1954.tb01243.x 13190171

[B3] Berlyne D. E. (1970). Novelty, complexity, and hedonic value. *Percept. Psychophys.* 8 279–286. 10.3758/BF03212593

[B4] Berlyne D. E. (1971). *Aesthetics and Psychobiology.* New York, NY: Appleton-Century-Crofts.

[B5] Bharucha J. J. (1984). Event hierarchies, tonal hierarchies and assimilation: a reply to deutsch and dowling. *J. Exp. Psychol. Gen.* 113 421–425.

[B6] Bigand E. Poulin-Charronnat B. (2016). “Tonal cognition,” in *Oxford Handbook of Music Psychology Ian Cross*, 2nd Edn, eds Thaut M. Hallam S. (Oxford: OUP), 1–19.

[B7] Blood A. J. Zatorre R. J. (2001). Intensely pleasurable responses to music correlate with activity in brain regions implicated in reward and emotion. *Proc. Natl. Acad. Sci. U.S.A.* 98 11818–11823. 10.1073/pnas.191355898 11573015 PMC58814

[B8] Boulez P. (1972). *Werkstatt-Texte.* Berlin: Propyläen.

[B9] Bowden E. M. Jung-Beeman M. Fleck J. Kounios J. (2005). New approaches to demystifying insight. *Trends Cogn. Sci.* 9 322–328. 10.1016/j.tics.2005.05.012 15953756

[B10] Brattico E. (2015). “From pleasure to liking and back: bottom-up and top-down neural routes to the aesthetic enjoyment,” in *Art, Aesthetics, and the Brain*, eds Huston J. P. Nadal M. Mora F. Agnati L. F. Conde C. J. C. (Oxford: Oxford University Press), 303–318.

[B11] Brattico E. (2021). “The empirical aesthetics of music,” in *The Oxford Handbook of Empirical Aesthetics*, eds Nadal M. Vartanian O. (Oxford: Oxford University Press), 1–38.

[B12] Brattico E. Pearce M. T. (2013). The neuroaesthetics of music. *Psychol. Aesthetics Creat. Arts* 7 48–61. 10.1037/a0031624

[B13] Brattico E. Bogert B. Alluri V. Tervaniemi M. Eerola T. Jacobsen T. (2016). It’s sad but i like it: The neural dissociation between musical emotions and liking in experts and laypersons. *Front. Hum. Neurosci.* 9:676. 10.3389/fnhum.2015.00676 26778996 PMC4701928

[B14] Bregman A. (1990). *Auditory Scene Analysis: the Perceptual Organization of Sound.* Cambridge, MA: MIT Press.

[B15] Brincker M. (2015). “The aesthetic stance – on the conditions and consequences of becoming a beholder,” in *Aesthetics and the Embodied Mind: Beyond Art Theory and the Cartesian Mind- Body Dichotomy Contributions to Phenomenology*, ed. Scarinzi A. (Berlin: Springer), 117–138. 10.1007/978-94-017-9379-7_8

[B16] Cacioppo J. T. Petty R. E. (1982). The need for cognition. *J. Pers. Soc. Psychol.* 42 116–131. 10.1037/0022-3514.42.1.116

[B17] Cacioppo J. T. Petty R. E. Feinstein J. A. Blair W. Jarvis G. (1996). Dispositional differences in cognitive motivation: the life and times of individuals varying in need for cognition. *Psychol. Bull.* 119 197–253.

[B18] Cheung V. K. M. Harrison P. M. C. Meyer L. Pearce M. T. Haynes J.-D. Koelsch S. (2019). Uncertainty and surprise jointly predict musical pleasure and amygdala, hippocampus, and auditory cortex activity. *Curr. Biol.* 29 4084–4092.e4. 10.1016/j.cub.2019.09.067 31708393

[B19] Clark A. (2013). Whatever next? Predictive brains, situated agents, and the future of cognitive science. *Behav. Brain Sci.* 36 181–253. 10.1017/S0140525X12000477 23663408

[B20] Clarke E. F. (1999). “Rhythm and timing in music,” in *The Psychology Of Music*, ed. Deutsch D. (Cambridge, MA: Academic Press), 473–500.

[B21] Cuddy L. L. Cohen A. J. Mewhort D. J. K. (1981). Perception of structure in short melodic sequences. *J. Exp. Psychol. Hum. Percept. Perform.* 7 869–883. 10.1037//0096-1523.7.4.869 6457099

[B22] Cupchik G. C. Gebotys R. J. (1990). Interest and pleasure as dimensions of aesthetic response. *Empir. Stud. Arts* 8 1–14.

[B23] Daynes H. (2011). Listeners’ perceptual and emotional responses to tonal and atonal music. *Psychol. Music* 39 468–502. 10.1177/0305735610378182

[B24] Deliège I. (1989). A perceptual approach to contemporary musical forms. *Contemp. Music Rev.* 4 213–230. 10.1080/07494468900640301

[B25] Deliège I. Mélen M. (1997). “Cue abstraction in the representation of musical form,” in *Perception And Cognition Of Music*, eds Deliege I. Sloboda J. A. (Abingdon: Taylor and Francis), 359–382. 10.4324/9780203344262-28

[B26] Denham S. L. Winkler I. (2006). The role of predictive models in the formation of auditory streams. *J. Physiol. Paris* 100 154–170. 10.1016/j.jphysparis.2006.09.012 17084600

[B27] Deutsch D. (1984). Two issues concerning tonal hierarchies: comment on castellano, bharucha, and krumhansl. *J. Exp. Psychol. Gen.* 113 413–416. 10.1037//0096-3445.113.3.413 6237170

[B28] Deutsch D. (1999). *The Psychology Of Music.* Cambridge, MA: Academic Press.

[B29] Deutsch D. (2013). “Grouping mechanisms in music,” in *The Psychology of Music*, ed. Deutsch D. (Cambridge, MA: Elsevier Academic Press), 183–248. 10.1016/B978-0-12-381460-9.00006-7

[B30] Dibben N. (1994). The cognitive reality of hierarchic structure in tonal and atonal music. *Music Percept.* 12 1–25.

[B31] Dibben N. (1999). The perception of structural stability in atonal music: the influence of salience, stability, horizontal motion, pitch commonality, and dissonance. *Perception* 16 265–294. 10.2307/40285794

[B32] Dibelius U. (1998). *Moderne Musik Nach 1945.* München: Piper.

[B33] Dowling W. J. Kwak S. Andrews M. W. (1995). The time course of recognition of novel melodies. *Percept. Psychophys.* 57 136–149. 10.3758/bf03206500 7885812

[B34] Dykstra A. R. Cariani P. A. Gutschalk A. (2017). A roadmap for the study of conscious audition and its neural basis. *Philos. Trans. R. Soc. B Biol. Sci.* 372:20160103. 10.1098/rstb.2016.0103 28044014 PMC5206271

[B35] Eerola T. Vuoskoski J. K. Peltola H. R. Putkinen V. Schäfer K. (2018). An integrative review of the enjoyment of sadness associated with music. *Phys. Life Rev.* 25 100–121. 10.1016/j.plrev.2017.11.016 29198528

[B36] Feist G. J. Brady T. R. (2004). Openness to experience, non-conformity, and the preference for abstract art. *Empir. Stud. Arts* 22 77–89. 10.2190/Y7CA-TBY6-V7LR-76GK 22612255

[B37] Fenner D. E. W. (1996). *The Aesthetic Attitude.* Atlantic Highlands, NJ: Humanities Press.

[B38] Fenner D. E. W. (1998). “Aesthetic Attitude,” in *Encyclopedia Of Aesthetics*, ed. Kelly M. (New York, NY: Oxford University Press).

[B39] Friston K. J. (2009). The free-energy principle: a rough guide to the brain? *Trends Cogn. Sci.* 13 293–301. 10.1016/j.tics.2009.04.005 19559644

[B40] Friston K. J. (2010). The free-energy principle: a unified brain theory? *Nat. Rev. Neurosci.* 11 127–138. 10.1038/nrn2787 20068583

[B41] Friston K. J. Kiebel S. (2009). Cortical circuits for perceptual inference. *Neural Netw.* 22 1093–1104. 10.1016/j.neunet.2009.07.023 19635656 PMC2796185

[B42] Garrido M. I. Sahani M. Dolan R. J. (2013). Outlier responses reflect sensitivity to statistical structure in the human brain. *PLoS Comput. Biol.* 9:e1002999. 10.1371/journal.pcbi.1002999 23555230 PMC3610625

[B43] Gazzaniga M. S. Ivry R. B. Mangun G. R. (2010). *Cognitive Neuroscience; the Biology of the Mind*, 4th Edn. New York, NY: W.W. Norton.

[B44] Gold B. P. Pearce M. T. Mas-Herrero E. Dagher A. Zatorre R. J. Zatorre R. J. (2019). Predictability and uncertainty in the pleasure of music: a reward for learning? *J. Neurosci.* 39 9397–9409. 10.1523/JNEUROSCI.0428-19.2019 31636112 PMC6867811

[B45] Graf L. K. M. Landwehr J. R. (2017). Aesthetic pleasure versus aesthetic interest: the two routes to aesthetic liking. *Front. Psychol.* 8:15. 10.3389/fpsyg.2017.00015 28194119 PMC5276863

[B46] Grant M. J. (2001). *Serial Music, Serial Aesthetics: Compositional Theory in Post-War Europe.* Cambridge: Cambridge University Press.

[B47] Griffiths P. (2001). *Serialism. Grove Music Online* (Oxford: Oxford University Press). 1–16.

[B48] Gross J. Pitts S. (2016). Audiences for the contemporary arts: Exploring varieties of participation across art forms in Birmingham, UK. *Participations* 13 4–23.

[B49] Gutschalk A. Micheyl C. Oxenham A. J. (2008). Neural correlates of auditory perceptual awareness under informational masking. *PLoS Biol.* 6:1156–1165. 10.1371/journal.pbio.0060138 18547141 PMC2422852

[B50] Haumann N. T. Lumaca M. Kliuchko M. Santacruz J. L. Vuust P. Brattico E. (2021). Extracting human cortical responses to sound onsets and acoustic feature changes in real music, and their relation to event rate. *Brain Res.* 1754 147248. 10.1016/j.brainres.2020.147248 33417893

[B51] Heilbron M. Chait M. (2018). Great expectations: is there evidence for predictive coding in auditory cortex? *Neuroscience* 389 54–73. 10.1016/j.neuroscience.2017.07.061 28782642

[B52] Helmholtz H. L. F. V. (1954). *On the Sensations of Tone as a Physiological Basis for the Theory of Music*, ed. Elli A. J. (New York, NY: Dover Publications). (original work published in 1863).

[B53] Hiekel P. (2016). “Neue musik,” in *Lexikon Neue Musik*, eds Hiekel P. Utz C. (Stuttgart:), 434–444.

[B54] Hodges D. A. (2016). “The Neuroaesthetics of Music,” in *Oxford Handbook of Music Psychology*, eds Hallam S. Cross I. Thaut M. (Oxford: Oxford University Press), 10.1093/oxfordhb/9780198722946.013.20

[B55] Hsu Y. Le Bars S. Hämäläinen J. A. Waszak F. (2015). Distinctive representation of mispredicted and unpredicted prediction errors in human electroencephalography. *J. Neurosci.* 35 14653–14660. 10.1523/JNEUROSCI.2204-15.2015 26511253 PMC6605467

[B56] Huron D. (2006). *Sweet Anticipation: Music And The Psychology Of Expectation.* Cambridge, MA: MIT Press.

[B57] Imberty M. (1993). How do we perceive atonal music? Suggestions for a theoretical approach. *Contemp. Music Rev.* 9 325–337.

[B58] Jepma M. Verdonschot R. G. van Steenbergen H. Rombouts S. A. R. B. Nieuwenhuis S. (2012). Neural mechanisms underlying the induction and relief of perceptual curiosity. *Front. Behav. Neurosci.* 6:5. 10.3389/fnbeh.2012.00005 22347853 PMC3277937

[B59] Jones M. R. (2015). “Musical time,” in *Oxford Handbook Music Psychology*, eds Hallam S. Cross I. Thaut M. (Oxford: Oxford University).

[B60] Jones M. R. (2019). *Time Will Tell – A Theory of Dynamic Attending.* New York, NY: Oxford University Press.

[B61] Jung-Beeman M. Bowden E. M. Haberman J. Frymiare J. L. Arambel-Liu S. Greenblatt R. (2004). Neural activity when people solve verbal problems with insight. *PLoS Biol.* 2:500–510. 10.1371/journal.pbio.0020097 15094802 PMC387268

[B62] Juslin P. N. (2013). From everyday emotions to aesthetic emotions: Towards a unified theory of musical emotions. *Phys. Life Rev.* 10 235–266. 10.1016/j.plrev.2013.05.008 23769678

[B63] Juslin P. N. Isaksson S. (2014). Subjective criteria for choice and aesthetic judgment of music: a comparison of psychology and music students. *Res. Stud. Music Educ.* 36 179–198. 10.1177/1321103X14540259

[B64] Kemp G. (1999). The aesthetic attitude. *Br. J. Aesthet.* 39 392–399.

[B65] Kidd C. Hayden B. Y. (2015). The psychology and neuroscience of curiosity. *Neuron* 88 449–460. 10.1016/j.neuron.2015.09.010 26539887 PMC4635443

[B66] Kliuchko M. Brattico E. Gold B. P. Tervaniemi M. Bogert B. Toiviainen P. (2019). Fractionating auditory priors: a neural dissociation between active and passive experience of musical sounds. *PLoS One* 14:e0216499. 10.1371/JOURNAL.PONE.0216499 31051008 PMC6499420

[B67] Koelsch S. (2012). *Brain and Music.* West Sussex: Wiley-Blackwell.

[B68] Koelsch S. (2014). Brain correlates of music-evoked emotions. *Nat. Rev. Neurosci.* 15 170–180. 10.1038/nrn3666 24552785

[B69] Koelsch S. Vuust P. Friston K. J. (2019). Predictive processes and the peculiar case of music. *Trends Cogn. Sci.* 23 63–77. 10.1016/J.TICS.2018.10.006 30471869

[B70] Kostka S. Santa M. (2018). *Materials and Techniques of Post-Tonal Music*, 5th Edn. New York, NY: Routledge.

[B71] Kounios J. Beeman M. (2009). The aha! moment: the cognitive neuroscience of insight. *Curr. Dir. Psychol. Sci.* 18 210–216. 10.1111/j.1467-8721.2009.01638.x

[B72] Kramer J. D. (1989). *The Time of Music.* New York, NY: Schirmer Books.

[B73] Kraus N. (2020). The joyful reduction of uncertainty: music perception as a window to predictive neuronal processing. *J. Neurosci.* 40 2790–2792. 10.1523/JNEUROSCI.0072-20.2020 32238483 PMC7117902

[B74] Krumhansl C. L. Cuddy L. L. (2010). “A theory of tonal hierarchies in music,” in *Music Perception, Springer Handbook of Auditory Research*, eds Jones M. R. Fay R. R. Popper A. N. (Berlin: Springer), 51–87. 10.1007/978-1-4419-6114-3_3

[B75] Krumhansl C. L. Sandell G. J. Sergeant D. C. (1987). The perception of tone hierarchies and mirror forms in twelve-tone serial music. *Music Percept.* 5 31–78.

[B76] Lehne M. Koelsch S. (2015). Toward a general psychological model of tension and suspense. *Front. Psychol.* 6:79. 10.3389/fpsyg.2015.00079 25717309 PMC4324075

[B77] Lerdahl F. (1989). Atonal prolongational structure. *Contemp. Music Rev.* 4 65–87. 10.1080/07494468900640211

[B78] Lerdahl F. (1992). Cognitive constraints on compositional systems. *Contemp. Music Rev.* 6 97–121. 10.1080/07494469200640161

[B79] Lerdahl F. (2019). *Composition And Cognition: Reflections on Contemporary Music and the Musical Mind.* Berkeley, CA: University of California Press.

[B80] Lerdahl F. Jackendoff R. (1983). *A Generative Theory Of Tonal Music.* Cambridge, MA: MIT Press.

[B81] Levinson J. (2009). The aesthetic appreciation of music. *Br. J. Aesthet.* 49 415–425. 10.1093/aesthj/ayp043

[B82] London J. (2004). *Hearing in Time: Psychological Aspects of Musical Meter.* Oxford: Oxford University Press.

[B83] Loui P. Wessel D. (2006). “Acquiring new musical grammars: a statistical learning approach,” in *Proceedings of the 9th International Conference on Music Perception and Cognition*, eds Baroni M. Addessi A. R. Caterina R. Costa M. (Bologna: ICMPC-ESCOM), 1009–1017.

[B84] Maletz H. (2011). *Leidenschaft? Neue Musik. Über Klänge, Laute, Zeichen Bis Zu Jazz und Pop.* Münster: Lit Verlag.

[B85] Marin M. (2020). “The role of collative variables in aesthetic experiences,” in *The Oxford Handbook of Empirical Aesthetics*, eds Nadal M. Vartanian O. (Oxford: Oxford University Press).

[B86] Marin M. M. Lampatz A. Wandl M. Leder H. (2016). Berlyne revisited: evidence for the multifaceted nature of hedonic tone in the appreciation of paintings and music. *Front. Hum. Neurosci.* 10:536. 10.3389/fnhum.2016.00536 27867350 PMC5095118

[B87] Mayring P. (2014). *Qualitative Content Analysis Theoretical Foundation, Basic Procedures and Software Solution.* Klagenfurt: Beltz Verlag.

[B88] McLain D. L. (2009). Evidence of the properties of an ambiguity tolerance measure: the multiple stimulus types ambiguity tolerance scale-ii (mstat-ii). *Psychol. Rep.* 105 975–988. 10.2466/PR0.105.3.975-988 20099561

[B89] Mencke I. Omigie D. Wald-Fuhrmann M. Brattico E. (2019). Atonal music: can uncertainty lead to pleasure? *Front. Neurosci.* 12:979. 10.3389/FNINS.2018.00979 30670941 PMC6331456

[B90] Mencke I. Quiroga-Martinez D. R. Omigie D. Schwarzacher F. Haumann N. T. Michalareas G. (2021). Prediction under uncertainty: dissociating sensory from cognitive expectations in highly uncertain musical contexts. *Brain Res.* 1773:147664. 10.1016/j.brainres.2021.147664 34560052

[B91] Mencke I. Seibert C. Brattico E. Wald-Fuhrmann M. (2022). Comparing the aesthetic experience of classic-romantic and contemporary classical music: An interview study. *Psychol. Music* 10.1177/03057356221091312

[B92] Menninghaus W. Wagner V. Hanich J. Wassiliwizky E. Jacobsen T. Koelsch S. (2017). The distancing-embracing model of the enjoyment of negative emotions in art reception. *Behav. Brain Sci.* 40 1–63. 10.1017/S0140525X17000309 28215214

[B93] Meyer L. B. (1956). *Emotion And Meaning In Music.* Chicago, IL: University of Chicago Press.

[B94] Morgan E. Fogel A. Nair A. Patel A. D. (2019). Statistical learning and Gestalt-like principles predict melodic expectations. *Cognition* 189 23–34. 10.1016/j.cognition.2018.12.015 30913527

[B95] Muth C. Carbon C. C. (2013). The aesthetic aha: on the pleasure of having insights into gestalt. *Acta Psychol. (Amst)* 144 25–30. 10.1016/j.actpsy.2013.05.001 23743342

[B96] Muth C. Hesslinger V. M. Carbon C. C. (2018). Variants of semantic instability (SeIns) in the arts: a classification study based on experiential reports. *Psychol. Aesthetics Creat. Arts* 12 11–23. 10.1037/aca0000113

[B97] Näätänen R. Paavilainen P. Rinne T. Alho K. (2007). The mismatch negativity (MMN) in basic research of central auditory processing: a review. *Clin. Neurophysiol.* 118 2544–2590. 10.1016/j.clinph.2007.04.026 17931964

[B98] Neuloh G. Curio G. (2004). Does familiarity facilitate the cortical processing of music sounds? *Neuroreport* 15 2471–2475. 10.1097/00001756-200411150-00008 15538177

[B99] Nusbaum E. C. Silvia P. J. (2011). Shivers and timbres: personality and the experience of chills from music. *Soc. Psychol. Personal. Sci.* 2 199–204. 10.1177/1948550610386810

[B100] Ockelford A. Sergeant D. (2012). Musical expectancy in atonal contexts: musicians’ perception of “antistructure.”. *Psychol. Music* 41 139–174. 10.1177/0305735612442582

[B101] Omigie D. (2015). Dopamine and epistemic curiosity in music listening Dopamine and epistemic curiosity in music listening. *Cogn. Neurosci.* 6 222–224. 10.1080/17588928.2015.1051013 26073880

[B102] Omigie D. Ricci J. (2022). Accounting for expressions of curiosity and enjoyment during music listening. *Psychol. Aesthet. Creat. Arts* 1–17. 10.1037/aca0000461 [Epub ahead of print].

[B103] Omigie D. Ricci J. (2021). Curiosity emerging from the perception of change in music. *Empir. Stud. Arts* 40 296–316. 10.1177/02762374211059460

[B104] Ordoñana J. A. Laucirica A. (2017). Structural segmentation of toru takemitsu’s piece, itinerant, by advanced level music graduate students. *Iperception* 8 1–17. 10.1177/2041669517705387 28515863 PMC5423594

[B105] Pearce M. T. (2018). Statistical learning and probabilistic prediction in music cognition: mechanisms of stylistic enculturation. *Ann. N. Y. Acad. Sci.* 1423 378–395. 10.1111/nyas.13654 29749625 PMC6849749

[B106] Pearce M. T. Wiggins G. A. (2012). Auditory expectation: the information dynamics of music perception and cognition. *Top. Cogn. Sci.* 4 625–652. 10.1111/j.1756-8765.2012.01214.x 22847872

[B107] Perlovsky L. I. (2010). Musical emotions: functions, origins, evolution. *Phys. Life Rev.* 7 2–27. 10.1016/j.plrev.2009.11.001 20374916

[B108] Quiroga-Martinez D. R. Hansen N. C. Højlund A. Pearce M. T. Brattico E. Vuust P. (2019). Reduced prediction error responses in high-as compared to low-uncertainty musical contexts. *Cortex* 120 181–200. 10.1016/j.cortex.2019.06.010 31323458

[B109] Quiroga-Martinez D. R. Hansen N. C. Højlund A. Pearce M. T. Brattico E. Vuust P. (2020a). Musical prediction error responses similarly reduced by predictive uncertainty in musicians and non-musicians. *Eur. J. Neurosci.* 51 2250–2269. 10.1111/ejn.14667 31891423

[B110] Quiroga-Martinez D. R. Hansen N. C. Højlund A. Pearce M. T. Brattico E. Vuust P. (2020b). Decomposing neural responses to melodic surprise in musicians and non-musicians: evidence for a hierarchy of predictions in the auditory system. *Neuroimage* 215:116816. 10.1016/j.neuroimage.2020.116816 32276064

[B111] Reber R. Schwarz N. Winkielman P. (2004). Processing fluency and aesthetic pleasure: is beauty in the perceiver’s processing experience? *Pers. Soc. Psychol. Rev.* 8 364–382. 10.1207/s15327957pspr0804_3 15582859

[B112] Reybrouck M. (2015). Music as environment: an ecological and biosemiotic approach. *Behav. Sci. (Basel)* 5 1–26. 10.3390/bs5010001 25545707 PMC4384059

[B113] Rohrmeier M. A. Koelsch S. (2012). Predictive information processing in music cognition. A critical review. *Int. J. Psychophysiol.* 83 164–175. 10.1016/j.ijpsycho.2011.12.010 22245599

[B114] Rosch E. (1975). Cognitive reference points. *Cogn. Psychol.* 7 532–547.

[B115] Ross S. Hansen N. C. (2016). Dissociating prediction failure: considerations from music perception. *J. Neurosci.* 36 3103–3105. 10.1523/JNEUROSCI.0053-16.2016 26985022 PMC6705525

[B116] Sachs M. E. Damasio A. Habibi A. (2015). The pleasures of sad music: a systematic review. *Front. Hum. Neurosci.* 9:404. 10.3389/fnhum.2015.00404 26257625 PMC4513245

[B117] Saklofske D. H. (1975). Aesthetic complexity and exploratory behavior. *Percept. Mot. Skills* 41 363–368. 10.2466/pms.1975.41.2.363 1187292

[B118] Salimpoor V. N. Benovoy M. Larcher K. Dagher A. Zatorre R. J. (2011). Anatomically distinct dopamine release during anticipation and experience of peak emotion to music. *Nat. Neurosci.* 14 257–262. 10.1038/nn.2726 21217764

[B119] Salimpoor V. N. Zald D. H. Zatorre R. J. Dagher A. McIntosh A. R. (2015). Predictions and the brain: how musical sounds become rewarding. *Trends Cogn. Sci.* 19 86–91. 10.1016/j.tics.2014.12.001 25534332

[B120] Schulze K. Dowling W. J. Tillmann B. (2012). Working memory for tonal and atonal sequences during a forward and a backward recognition task. *Music Percept.* 61 137–170. 10.1525/rep.2008.104.1.92.This

[B121] Schwartenbeck P. FitzGerald T. Dolan R. J. Friston K. J. (2013). Exploration, novelty, surprise, and free energy minimization. *Front. Psychol.* 4:710. 10.3389/fpsyg.2013.00710 24109469 PMC3791848

[B122] Shen W. Yuan Y. Liu C. Luo J. (2016). In search of the “Aha!” experience: elucidating the emotionality of insight problem-solving. *Br. J. Psychol.* 107 281–298. 10.1111/bjop.12142 26184903

[B123] Silvia P. J. (2008). Interest – the curious emotion. *Curr. Dir. Psychol. Sci.* 17 57–60. 10.1111/j.1467-8721.2008.00548.x

[B124] Snyder B. (2000). *Music and Memory: An Introduction.* Cambridge, MA: MIT Press.

[B125] Snyder B. (2008). “Memory for music,” in *Oxford Handbook Music Psychology*, eds Hallam S. Cross I. Thaut M. (Oxford: Oxfords University), 1–17. 10.1093/oxfordhb/9780199298457.013.0010

[B126] Sohoglu E. Chait M. (2016). Detecting and representing predictable structure during auditory scene analysis. *Elife* 5:e19113. 10.7554/eLife.19113 27602577 PMC5014546

[B127] Southwell R. Chait M. (2018). Enhanced deviant responses in patterned relative to random sound sequences. *Cortex* 109 92–103. 10.1016/j.cortex.2018.08.032 30312781 PMC6259587

[B128] Southwell R. Baumann A. Gal C. Barascud N. Friston K. J. Chait M. (2017). Is predictability salient? A study of attentional capture by auditory patterns. *Philos. Trans. R. Soc. B Biol. Sci.* 372:20160105. 10.1098/rstb.2016.0105 28044016 PMC5206273

[B129] Stockhausen K. (1954). *Klavierstücke I-IV.* London: Universal Edition.

[B130] Stockhausen K. (1963). *Texte zur Elektronischen und Instrumentalen Musik, Aufsätze 1952-1962*, Vol. 1. Köln: DuMont.

[B131] Subramaniam K. Kounios J. Parrish T. B. Jung-Beeman M. (2009). A brain mechanism for facilitation of insight by positive affect. *J. Cogn. Neurosci.* 21 415–432. 10.1162/jocn.2009.21057 18578603

[B132] Taruffi L. Koelsch S. (2014). The paradox of music-evoked sadness: an online survey. *PLoS One* 9: e110490. 10.1371/journal.pone.0110490 25330315 PMC4203803

[B133] Taruskin R. (2010). *Music In The Early Twentieth Century.* New York, NY: Oxford University Press.

[B134] Tillmann B. McAdams S. (2004). Implicit learning of musical timbre sequences: Statistical regularities confronted with acoustical (dis)similarities. *J. Exp. Psychol. Learn. Mem. Cogn.* 30 1131–1142. 10.1037/0278-7393.30.5.1131 15355141

[B135] Topolinski S. Reber R. (2010). Gaining insight into the “Aha” experience. *Curr. Dir. Psychol. Sci.* 19 402–405. 10.1177/0963721410388803

[B136] Utz C. (2016). “Wahrnehmung,” in *Lexikon Neue Musik*, eds Hiekel P. Utz C. (Kassel: Baerenreiter), 600–609.

[B137] Van de Cruys S. (2017). “Affective value in the predictive mind,” in *Philosophy and Predictive Processing*, eds Metzinger T. Wiese W. (Frankfurt am Main: MIND Group), 1–21. 10.15502/9783958573253

[B138] van Lieshout L. L. de Lange F. P. Cools R. (2020). Why so curious? Quantifying mechanisms of information seeking. *Curr. Opin. Behav. Sci.* 35 112–117. 10.1016/j.cobeha.2020.08.005

[B139] Vuust P. Frith C. (2008). Anticipation is the key to understanding music and the effects of music on emotion. *Behav. Brain Sci.* 31 599–600. 10.1017/S0140525X08005542

[B140] Vuust P. Kringelbach M. L. (2010). The pleasure of making sense of music. *Interdiscip. Sci. Rev.* 35 166–182. 10.1179/030801810X12723585301192

[B141] Vuvan D. T. Podolak O. M. Schmuckler M. A. (2014). Memory for musical tones: the impact of tonality and the creation of false memories. *Front. Psychol.* 5:582. 10.3389/fpsyg.2014.00582 24971071 PMC4054327

[B142] Wald-Fuhrmann M. Egermann H. O’Neill K. Czepiel A. Weining C. Meier D. (2021). Music listening in classical concerts: theory, literature review, and research program. *Front. Psychol.* 12:638783. 10.3389/fpsyg.2021.638783 33986708 PMC8110713

[B143] Webb M. E. Little D. R. Cropper S. J. (2018). Once more with feeling: Normative data for the aha experience in insight and noninsight problems. *Behav. Res. Methods* 50 2035–2056. 10.3758/s13428-017-0972-9 29052169

[B144] Winkler I. Háden G. P. Ladinig O. Sziller I. Honing H. (2009). Newborn infants detect the beat in music. *Proc. Natl. Acad. Sci. U.S.A.* 106 2468–2471. 10.1073/pnas.0809035106 19171894 PMC2631079

[B145] Wörner K. H. (1973). *Stockhausen – Life and Work.* Berkeley, CA: University of California Press.

[B146] Wundt W. (1896). *Grundriss Der Psychologie.* Leipzig: Wilhelm Engelmann.

